# Pterostilbene induces apoptosis and cell cycle arrest in diffuse large B-cell lymphoma cells

**DOI:** 10.1038/srep37417

**Published:** 2016-11-21

**Authors:** Yuanyuan Kong, Gege Chen, Zhijian Xu, Guang Yang, Bo Li, Xiaosong Wu, Wenqin Xiao, Bingqian Xie, Liangning Hu, Xi Sun, Gaomei Chang, Minjie Gao, Lu Gao, Bojie Dai, Yi Tao, Weiliang Zhu, Jumei Shi

**Affiliations:** 1 Department of Hematology, Shanghai Tenth People’s Hospital, Tongji University School of Medicine, Shanghai, 200072, China; 2 CAS Key Laboratory of Receptor Research, Drug Discovery and Design Center, Shanghai Institute of Materia Medica, Chinese Academy of Sciences, Shanghai, 201203, China; 3 College of life science and technology, Tongji University, Shanghai, 200092, China

## Abstract

Diffuse large B-cell lymphoma (DLBCL) is the most common type of non-Hodgkin lymphoma (NHL). Pterostilbene, a natural dimethylated analog of resveratrol, has been shown to possess diverse pharmacological activities, including anti-inflammatory, antioxidant and anticancer properties. However, to the best of our knowledge, there has been no study of the effects of pterostilbene upon hematological malignancies. Herein, we report the antitumor activity and mechanism of pterostilbene against DLBCL cells both *in vitro* and *in vivo*. We found that pterostilbene treatment resulted in a dose-dependent inhibition of cell viability. In addition, pterostilbene exhibited a strong cytotoxic effect, as evidenced not only by reductions of mitochondrial membrane potential (MMP) but also by increases in cellular apoptotic index and reactive oxygen species (ROS) levels, leading to arrest in the S-phase of the cell cycle. Furthermore, pterostilbene treatment directly up-regulated p-p38MAPK and down-regulated p-ERK1/2. *In vivo*, intravenous administration of pterostilbene inhibited tumor development in xenograft mouse models. Overall, the results suggested that pterostilbene is a potential anti-cancer pharmaceutical against human DLBCL by a mechanism involving the suppression of ERK1/2 and activation of p38MAPK signaling pathways.

Worldwide, diffuse large B-cell lymphoma (DLBCL) represents the most common malignant lymphoma subtype of non-Hodgkin lymphoma (NHL), accounting for roughly 30–40% of all newly-diagnosed cases^1^. Patients with DLBCL have variable clinical presentation and respond differently to combined chemoimmunotherapy, ranging from cure to treatment failure, relapse/refractory disease, or death; overall survival can range from a few weeks to over 10 years until the disease is cured^2^. Based on this remarkable heterogeneity, gene expression profiling (GEP) studies have identified three molecularly-distinct subtypes: termed germinal center B-cell (GCB), activated B-cell (ABC) and primary mediastinal B-cell lymphoma subtype or unclassifiable subtype, which represent lymphomas derived from different stages of B-cell lymphoid differentiation^3^,^4^. Within the last decade, advances in novel therapeutic regimens have greatly improved our understanding of DLBCL and its treatment by radio- and/or immuno-, multi-agent chemotherapy, combined with or without stem cell transplantation. While lasting remission can be achieved with current modern cancer therapy, approximately 30–40% of patients will do not respond to currently-available treatment or will develop relapsed/refractory disease with resistance^5^.

Stilbenes, including resveratrol and pterostilbene, are a class of natural polyphenolic compounds that shows pleiotropic health benefits including anti-carcinogenic activities. Pterostilbene (trans-3, 5-dimethoxy-4′-hydroxystilbene), a dimethylether analogue of resveratrol, is found predominantly in blueberries and grapes. Blueberries contain up to 15 μg of pterostilbene per 100 g of berries^6^ and are considered a rich source of these naturally-occurring stilbenes. Compared to resveratrol, pterostilbene shows relatively better bioavailability profiles^7^ and longer half-life^8^ following ingestion, indicating that pterostilbene might hold greater potential for clinical applications. Indeed, pterostilbene exhibits a wide spectrum of biological functions including antineoplastic, anti-inflammatory, antioxidant, apoptotic, antiproliferative, antimicrobial and analgesic potential^9^. In recent years, the anticarcinogenic effects of pterostilbene have gained increasing attention from many researchers. Several studies have shown that pterostilbene exerts antiproliferative and apoptosis-inducing effects in both solid (such as liver, colon, prostate and breast cancers)^10^,^11^,^12^,^13^ and non-solid tumors (such as acute/chronic myelogenous leukemia and lymphoblastic leukemia)^14^,^15^,^16^. Tolomeo and colleagues^16^ discovered that pterostilbene induced apoptosis in sensitive and multi-drug resistant leukemia cell lines *in vitro*. However, the effects of pterostilbene activity in B-cell malignancies, especially against DLBCL cells, and the possible mechanisms, have not been fully elucidated.

In the present study, we examined the antitumor activities of pterostilbene in human DLBCL cell lines both *in vitro* and *in vivo*. Furthermore, we explored the molecular mechanism behind pterostilbene action in cancer cell apoptosis.

## Results

### Pterostilbene inhibits the growth in DLBCL cell lines

**Figure 1:**
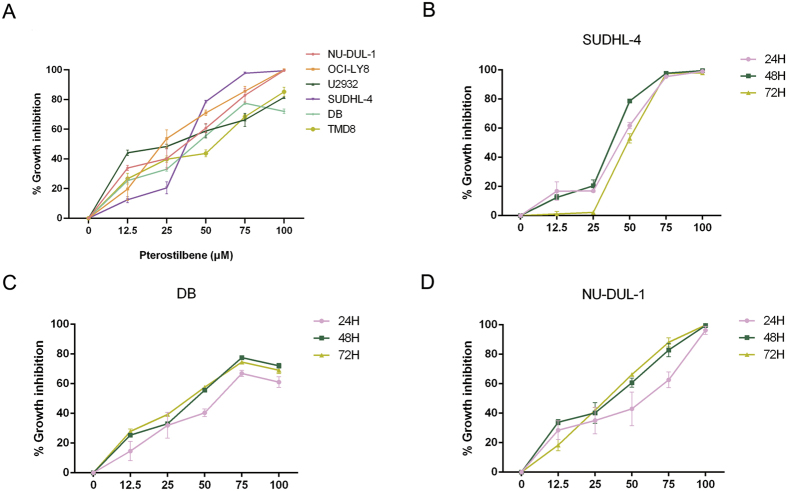
Pterostilbene inhibits cell proliferation in DLBCL cell lines. (**A**) Six DLBCL cell lines were treated with pterostilbene at various concentrations (12.5–100 μM) in serum-containing medium for 48 h, then cell viability was monitored by CCK-8 assay. (**B**) SUDHL-4, (**C**) DB and (**D**) NU-DUL-1 cells were treated with pterostilbene at various concentrations (12.5–100 μM) and time points (24, 48 and 72 h), then cell viability was monitored by CCK-8 assay. The results of the untreated group were taken as 0%.

To determine the efficacy of pterostilbene in DLBCL cells, we first evaluated cell viability using the CCK-8 assay in several DLBCL cell lines (OCI-LY8, SUDHL-4, DB, TMD8, U2932, and NU-DUL-1) treated with various concentrations of pterostilbene. The number of living cells was expressed as a percentage of the number of cells in untreated control cultures. After treatment for 48 h, the CCK8 assay showed that pterostilbene significantly inhibited cell proliferation in a dose-dependent manner (Fig. 1A). The calculated IC_50_ (50% cell growth inhibitory concentration) values were as follows: 27.22 (SUDHL-4), 32.11 (DB), 24.16 (NU-DUL-1), 24.12 (U2932), 26.64 (OCI-LY8), 32.19 μM (TMD8). To compared the cell viability in different subtypes, one or two of the same subtype were selected for further investigation. A time-course study of pterostilbene, however, showed that cancer cell growth was not inhibited in a time-dependent manner within the given concentration range (Fig. 1B–D).

### Pterostilbene provokes cell cycle arrest in DLBCL cells

**Figure 2:**
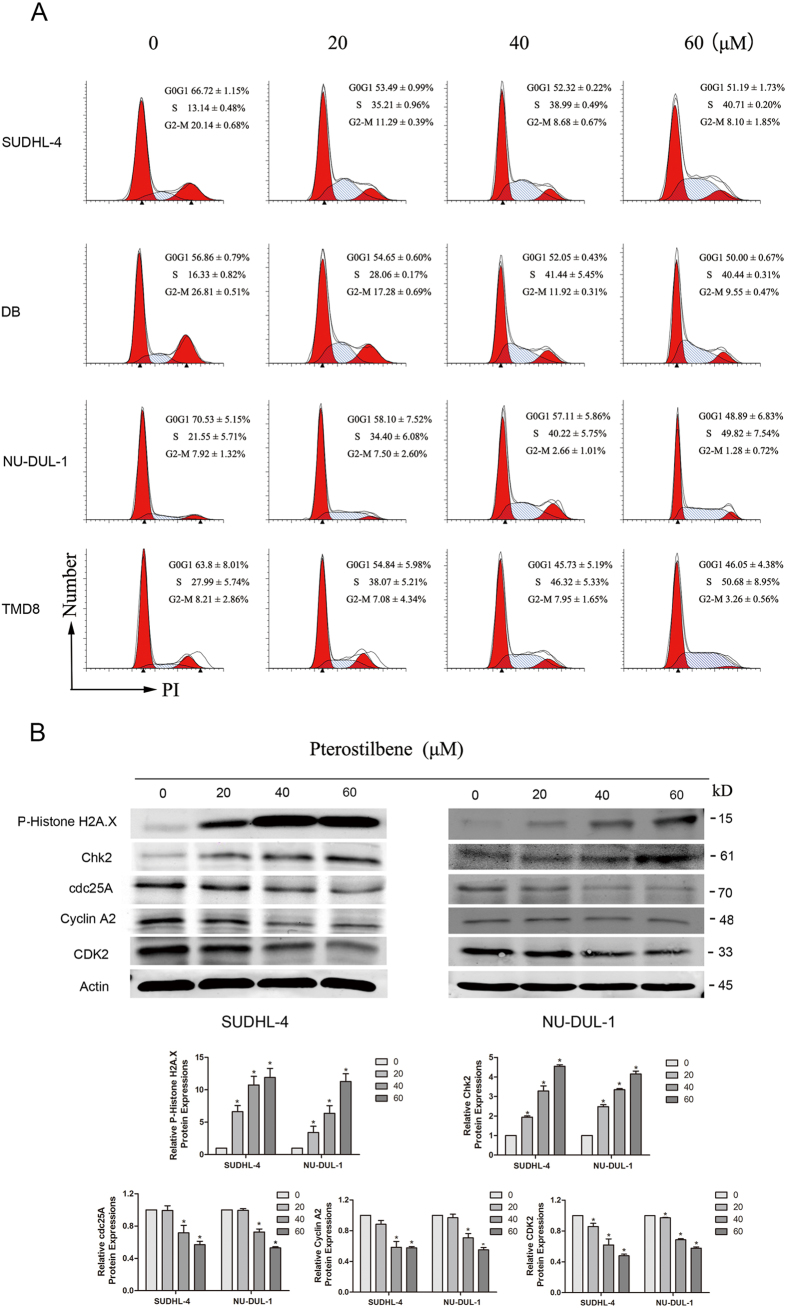
Pterostilbene induces cell cycle arrest in DLBCL cell lines. (**A**) Four DLBCL cell lines were treated for 24 h with pterostilbene (20, 40 and 60 μM) followed by propidium iodide staining and flow cytometric analysis. Values represent the mean ± SEM of three independent experiments performed in triplicate. (**B**) After treatment as in A, proteins were extracted from cultured SUDHL-4 and NU-DUL-1 cells and probed with appropriate dilutions of specific antibodies. Upper panels: Representative results of phospho-Histone H2A.X, chK2, Cyclin A2, CDK2, and cdc25A protein levels were as determined by a Western blot analysis. Lower panel: Quantitative results of related protein levels, which were adjusted with the actin protein level and expressed as multiples of induction beyond its own control. *p < 0.05, compared to the vehicle control group.

In this study, we found that pterostilbene demonstrated significant cytotoxicity in DLBCL cells. Therefore, to investigate the mode of this inhibition, the effect of pterostilbene on cell cycle was investigated using flow cytometry. The results showed that cells treated with pterostilbene accumulated in S-phase of the cell cycle in four of the cell lines tested (Fig. 2A). To study the molecular mechanisms underlying pterostilbene-induced S-phase arrest, cells were seeded into six-well plates with pterostilbene (20, 40, or 60 μM) for 24 h, after which they were collected for protein extraction and western blot analysis. As shown in Fig. 2B, levels of phospho-histone H2A.X and Chk2 proteins were significantly increased in pterostilbene-treated DLBCL cells. Cdc25A is a critical convergence point with the DNA-damage checkpoint, so to explore the complex network connecting cdc25A and CDK activity is essential. We examined protein levels of cdc25A, CDK2 and Cyclin A2, three known proteins that were found to decrease in pterostilbene-treated DLBCL cells.

### Pterostilbene induces apoptosis in DLBCL cells

**Figure 3:**
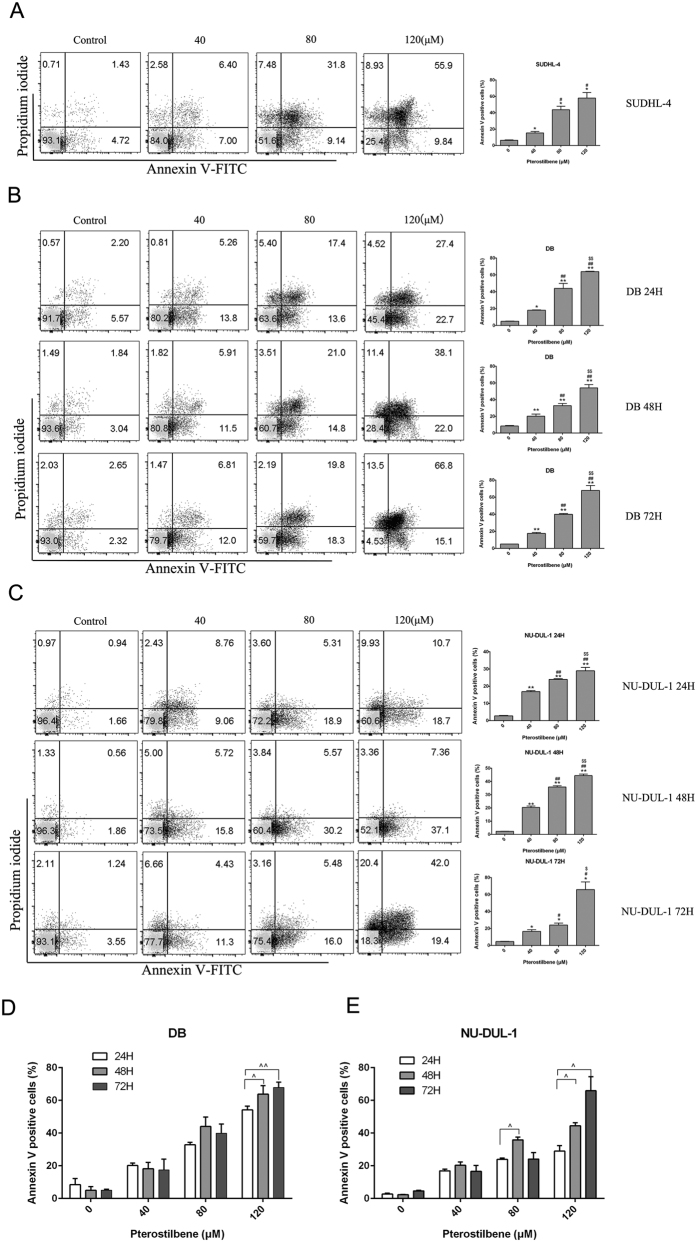
Pterostilbene increases apoptosis in DLBCL cell lines. (**A**) SUDHL-4 cells were treated with various concentrations of pterostilbene (40, 80 and 120 μM) for 48 h, then cell viability was analyzed using flow cytometry. (**B**) DB and (**C**) NU-DUL-1 cells were treated with pterostilbene (40, 80 and 120 μM) at different time points (24, 48 and 72 h). Apoptosis was detected using a FITC-Annexin V/PI staining kit and examined by flow cytometry. The apoptotic index was expressed as the number of apoptotic cells/total number of cells counted × 100%. Columns represent the average percentage of Annexin V positive cells from three independent experiments, which are shown as the means ± SEM. *p < 0.05, compared with control groups; **p < 0.01, compared with control groups; ^#^*p* < 0.05, compared with the pterostilbene 40 μM group; ^##^*p* < 0.01, compared with the pterostilbene 40 μM group; ^$$^*p* < 0.01, compared with the pterostilbene 80 μM group. (**D**) DB and (**E**) NU-DUL-1 cells were exposed to pterostilbene at different time points (24, 48 and 72 h) after which the percentage of apoptotic cells was determined by Annexin V analysis. Data show means ± SEM. *P* values were calculated using one-way ANOVA. ^*p* < 0.05, compared with the pterostilbene 24 h group; ^^*p* < 0.05, compared with the pterostilbene 24 h group.

The apoptotic procedure is executed by a number of highly-conserved caspases, and modulation of the mechanisms of caspase activation and suppression is a critical molecular target in chemoprevention, since these processes lead to apoptosis^17^. To study cell death triggered by pterostilbene in DLBCL cells, annexin-V/PI double staining was performed to evaluate the apoptotic effect using flow cytometric analysis. Compared with the control, the results indicated that pterostilbene increased the percentage of apoptotic cells in a concentration-dependent manner in the SUDHL-4 cell line (Fig. 3A). However, the action of pterostilbene on DLBCL cells was not time-dependent within the given concentration range as a whole (Fig. 3B–E). These results were consistent with the CCK-8 assay.

### Pterostilbene induces mitochondrial membrane potential and reactive oxygen species generation

**Figure 4:**
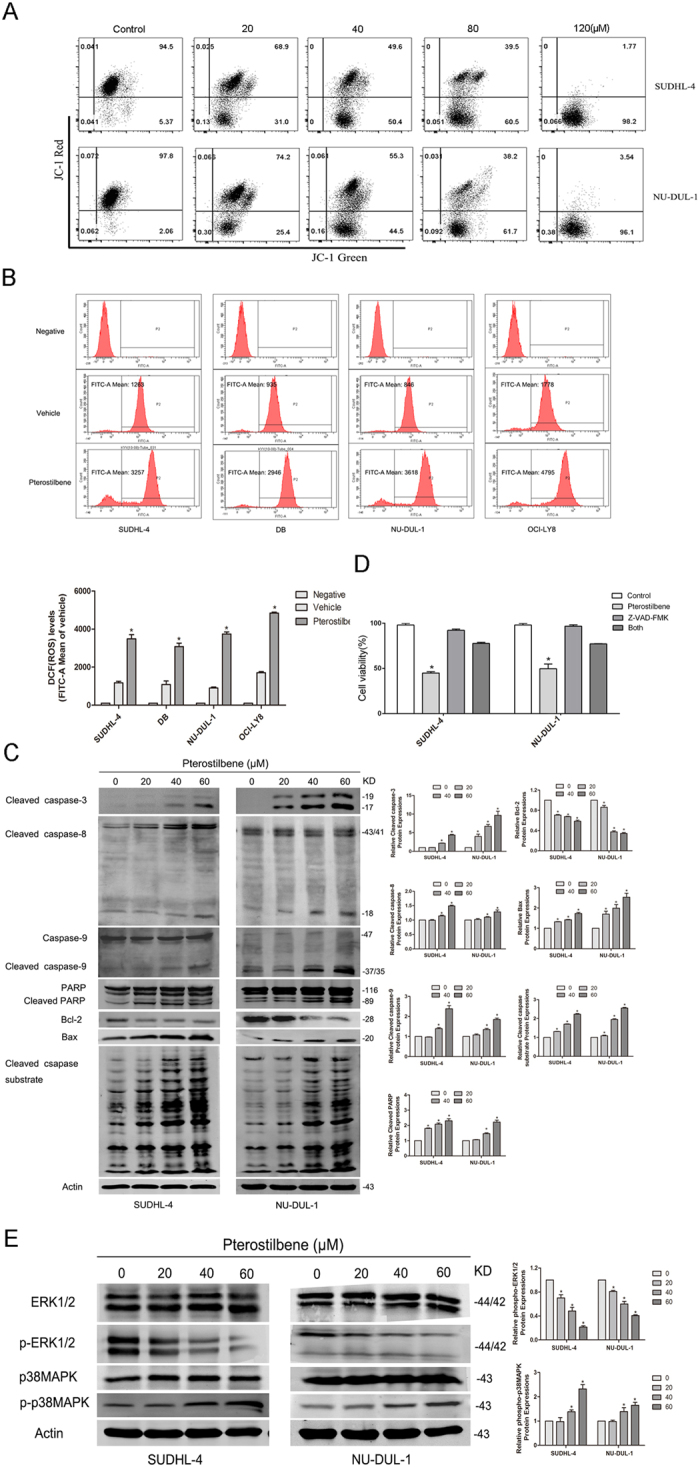
Pterostilbene activates caspase via ERK1/2 and p38MAPK signaling pathways. (**A**) After 24 h of drug exposure, cells were stained with JC-1 fluorescent dye, and mitochondrial membrane potential was detected by flow cytometry. (**B**) SUDHL-4, NU-DUL-1, DB and OCI-LY8 cells were treated with pterostilbene (60 μM) for 24 h and stained with DCFH-DA; then the level of ROS was detected by flow cytometry and data are presented as the mean fluorescence intensity. Values are presented as the means ± SEM of three independent experiments. *p < 0.05, compared to the vehicle control group. (**C**) After pterostilbene treatment for 48 h, the expression of cleaved caspase-3, cleaved caspase-8, caspase-9, cleaved caspase substrate, Bcl-2, Bax and PARP were monitored by western blotting. (**D**) SUDHL-4 and NU-DUL-1 cells were pretreated with or without 50 μM of pan-caspase inhibitor Z-VAD-FMK for 1 h and then exposed to 60 μM pterostilbene for 24 h. *p < 0.05. (**E**) SUDHL-4 and NU-DUL-1 cells were treated with pterostilbene (20, 40 or 60 μM) for 48 h, then the expression of phospho(p)-ERK1/2, ERK1/2, p-p38MAPK and p38MAPK were monitored by western blotting. Quantitative results of p-ERK1/2 and p-p38MAPK protein levels, which were adjusted with the total ERK1/2 and p38MAPK protein levels and expressed as multiples of induction beyond each respective control. Values represent the means ± SEM of three independent experiments. *p < 0.05, compared to the vehicle control group.

Collapse of mitochondrial integrity is an early step in the induction of apoptosis by the intrinsic pathway^18^. To investigate whether the apoptosis induced by pterostilbene involved mitochondrial depolarization, mitochondrial membrane potential (MMP) was measured using the JC-1 MMP detection kit. In healthy, non-apoptotic cells, the JC-1 dye accumulates as an aggregate in the mitochondrial matrix and this emits a strong red fluorescence. In unhealthy, apoptotic cells, due to the loss of membrane potential, JC-1 cannot aggregate within mitochondria and only exists in monomeric form in the cytoplasm producing green fluorescence. As shown in Fig. 4A, the result indicates that pterostilbene was capable of disrupting the MMP and, thus, of activating the intrinsic apoptosis pathway. Moreover, previous studies have reported that oxidative stress induced by pterostilbene originates from increasing levels of reactive oxygen species (ROS) in HL-60 cells^14^ and breast cancer cells^13^, and ROS are principal mediators of the ROS-dependent MMP apoptosis pathway. To investigate whether pterostilbene induces intracellular oxidation, we evaluated ROS levels in treated four DLBCL cell lines by flow cytometry using the specific oxidation-sensitive fluorescent dyes DCFH-DA. Treatment with pterostilbene (60 μM) for 24 h markedly increased ROS production. These findings support our hypothesis that the induced apoptosis after pterostilbene treatment is mediated through increasing ROS levels in DLBCL cells.

### Pterostilbene treatment resulted in caspase activation

To further explore the mechanisms underlying pterostilbene-induced apoptosis in DLBCL cells, caspases-3, −8, −9, cleavage of caspase substrate, PARP and mitochondrial apoptotic pathway-related proteins were detected by western blot analysis. Figure 4C shows that exposure of SUDHL-4 and NU-DUL-1 cells to pterostilbene (20, 40, or 60 μM) caused dose-dependent increases in caspases-3, −8, and −9, caspase substrate cleavage, as well as PARP cleavage. The reduced depolarization was further confirmed by down-regulated Bcl-2 and up-regulated Bax with pterostilbene treatment. Meanwhile, we also evaluated whether pterostilbene-induced cell death occurred through a caspase-dependent pathway. Pretreatment with the pan-caspase-inhibitor Z-VAD-FMK followed by CCK8 assay was used to monitor cell proliferation over 24 h. Our data showed that caspase inhibitor attenuated pterostilbene-induced inhibition of cell growth, consistent with our previous study (Fig. 4D).

### ERK1/2 and p38MAPK were modulated by pterostilbene-induced apoptosis

The phosphorylated forms of MAPK family members (ERK1/2 and p38MAPK) were assessed by western blot analysis in SUDHL-4 and NU-DUL-1 cells treated with pterostilbene (20, 40 or 60 μM) for 48 h. As shown in Fig. 4E, compared with control, treatment with pterostilbene for 48 h markedly decreased p-ERK1/2 and increased p-p38MAPK in a dose-dependent manner. Pterostilbene treatment did not change the total ERK1/2 and p38MAPK levels. Together, these data suggest that activation of p38MAPK and suppression of ERK1/2 contributes to cell apoptosis induced by pterostilbene treatment.

### Pterostilbene inhibits tumor growth *in vivo*

**Figure 5:**
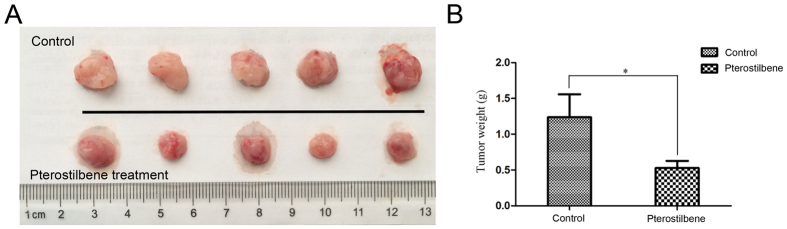
Pterostilbene inhibits the growth of implanted OCI-LY8 cells in a xenograft mouse model. OCI-LY8 human DLBCL cells (5 × 10^6^) were subcutaneously injected into the upper flank region of 6-week-old male nude mice. After the tumor size reached an approximate volume of 100 mm^3^, mice were randomly assigned into two groups: one group was intravenous DMSO/saline only (control), and the other group was administered pterostilbene by intravenous injection (30 mg/kg/2 days) for 20 days. (**A**) Tumor samples were collected and imaged using a high-definition digital camera. (**B**) Tumor weight was recorded. *p < 0.05.

Next, we examined the therapeutic efficacy of pterostilbene by treating male nude mice bearing human DLBCL tumor xenografts *in vivo*. We established OCI-LY8 DLBCL xenografts in 10 six-week-old male nude mice by injection into the upper flank region. Once the tumor size reached approximately 100 mm^3^, we treated the mice every other day by intravenous infusion both in the control group and pterostilbene group. During the experimental period, all mice were monitored to investigate whether pterostilbene treatment caused lethal toxicity, unhealthy symptoms or gross abnormalities. No evidence of tissue damage or noticeable side effects was observed during visible inspections and microscopic examination of individual organs (data not shown). After twenty days, pterostilbene markedly inhibited tumor growth (Fig. 5A) and tumor weight was also significantly inhibited (Fig. 5B). Our results strongly suggest that pterostilbene could be a novel promising agent for cancer treatment.

## Discussion

Diffuse large-B-cell lymphoma is a molecularly-heterogeneous disease^19^ and over 30% of patients will develop relapsed/refractory disease with the current standard treatment of rituximab and CHOP (cyclophosphamide, doxorubicin, vincristine and prednisolone) chemotherapy (R-CHOP)^20^. To improve the less favorable outcomes, evaluation and development of novel chemopreventive and/or chemotherapeutic agents are essential and challenging tasks.

Epidemiological studies have linked the benefits of a diet rich in fruits and vegetables in acting against a variety of diseases including human cancers. One such food source is berries, natural products which are abundant sources of antioxidant phytochemicals. Resveratrol and pterostilbene are precisely two phytoalexins found in plants with various health-promoting effects. Resveratrol, a polyphenol categorized as a phytoalexin^21^, is capable of protecting against various cancers. Indeed, recent studies showed that resveratrol exerts powerful antiproliferative and proapoptotic effects in solid and non-solid tumor cells *in vitro* and *in vivo*^22^,^23^,^24^,^25^. Compared to resveratrol, pterostilbene has higher oral bioavailability (80% versus 20%) and a longer half-life (105 minutes versus 14 minutes)^8^,^26^,^27^,^28^. A clinical trial demonstrated that pterostilbene is generally safe and well-tolerated for use in humans^29^. In addition, various molecules and signaling pathways are involved in the anti-tumor effects of pterostilbene. For example, pterostilbene induces both apoptosis and autophagy in bladder cancer cells^30^, and suppresses 12-O-tetradecanoylphorbol 13-acetate (TPA)-mediated cell invasion, migration and metastasis by decreasing MMP-9 activity in human hepatoma HepG2 cells^31^. However, the effects and mechanisms of pterostilbene on human DLBCL have not been elucidated.

In our study, we assessed the effects of pterostilbene on various cellular and molecular endpoints in the setting of DLBCL. First, we demonstrated that pterostilbene showed a dose-dependent cytotoxic effect on six human DLBCL cell lines, OCI-LY8, SUDHL-4, DB, TMD8, U2932, and NU-DUL-1 (Fig. 1A) with an approximate IC_50_ of 30 μM after 48 h. Pterostilbene-induced cell proliferation, in a concentration-dependent fashion, has also been observed in other hematological malignancies, including acute myeloid leukemia (AML)^14^ and MOLT4 human lymphoblastic leukemia^32^. In addition, we also found that pterostilbene-induced cell viability was not inhibited in a time-dependent manner in three DLBCL cell lines (SUDHL-4, DB and NU-DUL-1) within the setting concentration range. These results were consistent with those of flow cytometric analysis, suggesting that pterostilbene could reduce cell growth over a certain concentration range in a manner that was not time dependent. Other less-defined cell death mechanisms have been studied that appear not to require the caspase-dependent apoptosis pathway.

Uncontrolled cell proliferation is the hallmark of cancer and tumor cells are directly regulated by the cell cycle^33^. Hence, we evaluated the effect of pterostilbene on the cell cycle. Flow cytometric analysis revealed that more lymphoma cells were arrested in S-phase when incubated with different concentrations of the compound for 24 h. Similar results were previously reported in HL60 leukemia cells^16^, MCF7 breast cancer cells^13^ and T24 human bladder cancer cells^30^. However, the possible mechanism associated with DNA damage and repair caused by S-arrest required investigation. H2AX is a variant of the histone H2A family^34^ and phospho-H2AX plays a key role in DNA damage response and is essential for the assembly of DNA repair proteins in cell cycle progression^35^. Indeed, western blot analyses showed that levels of phospho-H2AX were increased after treatment with pterostilbene. Similarly, CHK2, a protein kinase that is an important mediator of the DNA damage checkpoint, phosphorylates a range of proteins involved in cell cycle control including cdc25A^36^. Western blot analyses showed that pterostilbene treatment down-regulated protein levels of cyclin A2, CDK2, and cdc25A and up-regulated the levels of Chk2 (Fig. 2B). These findings suggest that CHK2 expression is triggered by pterostilbene-induced DNA damage and cdc25A expression. Thus, the increase in H2AX and CHK2 provides insight into the mechanism of the effects of pterostilbene.

Apoptosis is a physiological process resulting in a highly-regulated, programmed form of cell death that is a normal part of growth and development in multicellular organisms. Chemical compounds that affect apoptotic pathways and eliminate cancer cells are considered promising anticancer drugs^14^. In this study, several hallmarks of apoptosis were detected in pterostilbene-treated DLBCL cells. In the annexin-V/PI co-staining assay, we observed that pterostilbene demonstrated a dose-dependent increase in SUDHL-4 cells (Fig. 3A). Similar results have been also been observed in other types of cancer cells such as the multidrug-resistant leukemia cells (HL60-R and K562-ADR) and Fas-ligand-resistant lymphoma cells (HUT78B1 and HUT78B3)^16^,^37^. Consistent with CCK-8 results, cancer cell growth was not inhibited in a time-dependent manner within the given concentration range after pterostilbene treatment.

It has been demonstrated that apoptosis involves loss of mitochondrial transmembrane potential, a mechanism that is decisive in physiological cell death. In our study, we detected the effect of pterostilbene on mitochondrial function. Our data demonstrated that pterostilbene causes cancer cell mitochondrial depolarization at the early stages of apoptosis (Fig. 4A). In addition, the increase in the mean DCFH–DA fluorescence intensity proved the accumulation of intracellular ROS generation causes oxidative stress-mediated cell death. In our study, we showed both an increase in ROS production and mitochondrial depolarization following treatment with pterostilbene, which indicates that the pterostilbene-induced apoptosis in DLBCL cells was mediated by the intrinsic (mitochondrial) apoptotic pathway, followed by activation of caspase activity triggering programmed cell death. These proteins were associated with the cleaved caspase-3, cleaved caspase-8, cleaved caspase substrate and caspase-9 occurring at 48 h and sequentially in SUDHL-4 and NU-DUL-1 cells exposed in a dose-dependent manner to pterostilbene. The anti-apoptotic mitochondrial protein Bcl-2 was decreased, while the pro-apoptotic mitochondrial protein Bax was increased by pterostilbene treatment in DLBCL cells. In addition, our data show that pterostilbene treatment of both cells caused the degradation of 116 kDa PARP into 89 kDa fragments in a dose-dependent manner. To further explore whether the activation of caspase cascades is necessary, a pan-caspase inhibitor, Z-VAD-FMK was added to two DLBCL cell lines (SUDHL-4 and NU-DUL-1). The results demonstrated that pretreatment with the pan-caspase inhibitor Z-VAD-FMK rescued pterostilbene-induced cell death, suggesting that apoptosis is induced by pterostilbene through the intrinsic caspase-dependent mechanism. This finding coincides with a previous study by Pan *et al*. on the apoptotic effect of pterostilbene on gastric carcinoma cells^38^. However, Tolomeo *et al*. found conflicting results when treating leukemia cells with pterostilbene^16^. They found that Z-VAD-FMK was not capable of inhibiting apoptosis when leukemia cells were exposed to both pterostilbene and its inhibitor. Further research into the biochemical mechanism of pterostilbene-mediated apoptosis is warranted.

The MAPK signaling pathway plays a central role in many cellular responses, such as cell proliferation, differentiation, migration and apoptosis^39^. MAPKs consist of three major subfamilies, including ERK1/2 and p38MAPK. Previous studies have indicated that the apoptosis induction of several tumor cells is associated with the MAPK signal transduction pathway^14^. In our study, we further investigated levels of MAPK-related proteins in SUDHL-4 and NU-DUL-1 cells treated with different concentrations of pterostilbene (20, 40, or 60 μM). Our results showed that pterostilbene was observed to decrease the level of ERK1/2 phosphorylation and increase p38MAPK phosphorylation, whereas total ERK1/2 and p38MAPK levels exhibited no change. Taken together, these results indicated that activation of p38MAPK and suppression of ERK1/2 play pivotal roles in pterostilbene-induced apoptosis of DLBCL cells via a caspase-dependent mechanism.

Several studies have revealed that dietary administration of pterostilbene inhibited the growth of colon tumor cells *in vivo*^40^,^41^. Paul *et al*. have previously reported that pterostilbene significantly reduced colon tumor multiplicity of aberrant crypt foci formation, lowered proliferating cell nuclear antigen and downregulated the expression of a cell proliferation marker after injection of the colon-specific carcinogen azoxymethane in rats. Meanwhile, our results showed that pterostilbene caused cytotoxic effects and apoptosis *in vitro*, and we further investigated the action of pterostilbene on DLBCL tumors in a xenograft mouse model. In our study, we found that intravenous administration of 30 mg/kg pterostilbene significantly inhibited tumor weight in male mice, suggesting that pterostilbene is of great interest for the prevention of at-risk patients.

In summary, the present study demonstrated the anticancer effect of pterostilbene in human DLBCL cancer cells *in vitro*. The activity was attributed to inhibition of cell viability, induction of apoptosis and ROS generation. Pterostilbene induced accumulation of lymphoma cells in S-phase of the cell cycle. MAPKs (ERK1/2 and p38MAPK) may play a key role in the chemopreventive effects of this polyphenolic compound in DLBCL. Furthermore, intravenous administration of pterostilbene was capable of inhibiting tumor growth in the nude mouse xenograft model. Our findings revealed that pterostilbene holds great promise for clinical evaluation in DLBCL patients. However, further investigations of the detailed molecular mechanism involved in pterostilbene-induced apoptosis are necessary.

## Materials and Methods

### Cells

The human SUDHL-4 and DB cell lines were purchased from the American Type Culture Collection (Manassas, VA, USA). NU-DUL-1 and OCI-LY8 cell lines were acquired from Professor Xiaoyan Zhou of the Department of Pathology, Fudan University Shanghai Cancer Center (Shanghai, China). TMD8 and U2932 cells were provided by Professor Dongsheng Xu of the Shanghai Tenth People’s Hospital, Tongji University of Medicine (Shanghai, China). The DLBCL cell lines SUDHL-4, DB, and OCI-LY8 belong to the GCB subtype, while the other three cell lines were the ABC subtype.

### Cell culture

Continuous neoplastic cells (SUDHL-4, DB, NU-DUL-1, TMD8) were grown in RPMI 1640 medium (Gibco, Life Technologies, Carlsbad, CA, USA) enriched with 10% fetal bovine serum (FBS; Gibco) and 1% penicillin–streptomycin (Gibco). U2932 cells were maintained in Dulbecco’s Modified Eagle’s Medium/Low Glucose (Gibco) containing 10% FBS and 1% penicillin–streptomycin. OCI-LY8 cells were maintained in Iscove’s Modified Dulbecco’s Medium (Gibco) containing 10% FBS and 1% penicillin–streptomycin. All cells were incubated in a humidified atmosphere of 5% CO_2_ at 37 °C.

### Reagents

A 40 mM pterostilbene stock solution was dissolved in dimethyl-sulfoxide (DMSO; Sigma, St Louis, MO, USA) and stored in the dark at −20 °C. Antibodies, specifically against cleaved caspase substrate, ERK1/2, phospho-ERK1/2, p38 MAPK, phospho-p38 MAPK, phospho-Histone H2A.X, cdc25A, CDK2, cleaved Caspase-3, cleaved Caspase-8, Caspase-9, Bax, Bcl-2, PARP, cleaved caspase substrate and β-actin (for western blot analysis), were purchased from Cell Signaling Technology (Danvers, MA, USA). Anti-cyclin A2 and anti-CHK2 antibodies were purchased from Epitomic (Burlingame, CA, USA). The pan-caspase inhibitor Z-VAD-FMK was obtained from Selleckchem (Houston, USA). Cell Counting Kit-8 (CCK-8) was purchased from Dojindo (Kumamoto, Japan). JC-1 Mitochondrial Membrane Potential Detection Kit was purchased from Beyotime Institute of Biotechnology (Shanghai, China).

### Cytotoxicity assay

Cells (2 × 10^5^ cells/mL) were plated into 96-well plates and treated with various concentrations of pterostilbene for 48 h in the incubator. At the end of the incubation, 10 μL of CCK-8 solution was added into each well of the plate and returned to the incubator for an additional 2 h at 37 °C. The absorbance was measured at 450 nm using a microplate reader.

### Cell cycle analysis

Cells (4 × 10^5^ cells/mL) were seeded into six-well culture plates, then treated with pterostilbene and harvested after 24 h. Harvested cells were washed with ice-cold phosphate buffered saline (PBS) and fixed with 70% ethanol at −20 °C overnight. The ethanol-fixed cells were washed with PBS, stained with 500 μL of 50 μg/mL propidium iodide (PI) at 37 °C for 15 min and assessed using a BD FACSCanto II flow cytometer (BD BioScience, San Jose, CA, USA).

### Analysis of cell apoptosis

Cell apoptosis assessment was performed using the double-staining method of the Annexin-V/PI apoptosis detection kit (BD Pharmingen, Franklin Lakes, NJ, USA). SUDHL-4, NU-DUL-1, TMD8 and DB cells were seeded into six-well plates and incubated for 48 h. According to the manufacturer’s instructions, cells were then stained with Annexin V-FITC/PI dye and analyzed immediately by flow cytometry. Apoptotic cells were identified as either Annexin V^+^/PI^−^ staining (early apoptosis) or Annexin V^+^/PI^+^ staining (late apoptosis).

### Mitochondrial membrane potential (MMP) measurement

The mitochondrial depolarization occurring in apoptosis was detected by flow cytometry using JC-1 staining following the manufacturer’s protocol (Mitochondrial Membrane Potential Detection Kit, Beyotime Institute of Biotechnology, Shanghai, China). Cells, adjusted to an approximate density of 1 × 10^6^ mL^–1^, were incubated at 37 °C, in 5% CO_2_ for 15 to 30 minutes in the presence of JC-1 (2 μM). Then, cells were suspended in warm phosphate-buffered saline (PBS) and analyzed on a flow cytometer with 488 nm excitation.

### ROS generation assessment

Cells were treated with pterostilbene (60 μM) for 24 h in the incubator. After treatment, cells were harvested and subsequently incubated with 10 μM 2′, 7′-dichlorodihydrofluorescein diacetate (DCFH-DA, Sigma) in serum-free cell culture medium at 37 °C for 20 min. After incubation, fluorescence intensity was monitored by fluorescence microscopy and flow cytometry, with 525 nm as the emission wavelength and 488 nm as the excitation wavelength.

### Western blot analysis

Cells were treated with different concentrations of pterostilbene. Then cytosolic proteins were extracted with lysis buffer (100 mM Tris-HCL, PH 6.8, 4% SDS, 20% glycerol). Equivalent amounts of total protein (30 μg per lane) were electrophoretically separated on a 10–12% sodium dodecyl sulfate-polyacrylamide gel (SDS-PAGE), transferred to a polyvinylidene difluoride membrane, blocked for 1 h with 5% defatted milk or 5% BSA, and incubated overnight at 4 °C with primary antibodies. Membranes were washed three times with Tween 20 (1:1000 dilution)-phosphate-buffered saline (PBS) and incubated for 60 min at room temperature with the appropriate secondary antibody (anti-rabbit or anti-mouse IgG). The protein signals were detected by the Odyssey two-color infrared laser imaging system (LICOR, Lincoln, NE, USA).

### Antitumor activity in a xenograft model

Tumor xenograft models were created using 6-week-old male nude mice (athymic, BALB/C nu/nu). Mice were purchased from Shanghai Laboratory Animal Center (SLAC, Shanghai, China) and housed in a standard laboratory with free access to water and food. They were kept in an air-conditioned room at 24 °C with a 12-hour light-dark cycle. Human OCI-LY8 cells (5 × 10^6^) suspended in 100 μL serum-free culture medium were injected into the upper flank region of male nude mice. After the tumor size reached an approximate volume of 100 mm^3^, ten mice were randomly divided into two groups: the vehicle group (DMSO/saline only) and pterostilbene-treated group (dissolved in DMSO/saline solution). Mice were administered pterostilbene by intravenous injection (30 mg/kg/2 days) for 20 days. The control group was intravenous DMSO/saline at the same time. All mice were sacrificed at the end of the experiment and the tumors were obtained and imaged. All animal-related procedures were approved by the Animal Care and Use Committee of The Tenth People’s Hospital of Shanghai, Tongji University. This research was approved by the Science and Technology Commission of Shanghai Municipality (ID: SYXK 2007–0006).

### Statistical Analysis

Data are expressed as means ± SEM of triplicate experiments (n = 3). Data comparisons among the experimental groups were performed using Student’s t-test. Significance of multiple comparisons were determined by one-way ANOVA with the SPSS v22.0 statistical analysis software (IBM Corp., Armonk, NY, USA). *p* < 0.05 was considered significant. All methods were performed in accordance with the guidelines and regulations of Shanghai Tongji University.

## Additional Information

**How to cite this article**: Kong, Y. *et al*. Pterostilbene induces apoptosis and cell cycle arrest in diffuse large B-cell lymphoma cells. *Sci. Rep.* **6**, 37417; doi: 10.1038/srep37417 (2016).

**Publisher’s note:** Springer Nature remains neutral with regard to jurisdictional claims in published maps and institutional affiliations.

## References

[b1] Sehn L. H. & Gascoyne R. D. Diffuse large B-cell lymphoma: optimizing outcome in the context of clinical and biologic heterogeneity. Blood 125, 22 (2015). 25499448 10.1182/blood-2014-05-577189

[b2] Rimsza L. M. . Loss of MHC class II gene and protein expression in diffuse large B-cell lymphoma is related to decreased tumor immunosurveillance and poor patient survival regardless of other prognostic factors: a follow-up study from the Leukemia and Lymphoma Molecular Profiling Project. Blood 103, 4251 (2004). 14976040 10.1182/blood-2003-07-2365

[b3] Hans C. P. Confirmation of the molecular classification of diffuse large B-cell lymphoma by immunohistochemistry using a tissue microarray. Blood 103, 275 (2004). 14504078 10.1182/blood-2003-05-1545

[b4] Alizadeh A. A. . Distinct types of diffuse large B-cell lymphoma identified by gene expression profiling. Nature 403, 503 (2000). 10676951 10.1038/35000501

[b5] Pasqualucci L. & Dalla-Favera R. SnapShot: diffuse large B cell lymphoma. Cancer Cell 25, 132 (2014). 24434215 10.1016/j.ccr.2013.12.012

[b6] Rimando A. M., Kalt W., Magee J. B., Dewey J. & Ballington J. R. Resveratrol, pterostilbene, and piceatannol in vaccinium berries. J Agric Food Chem 52, 4713 (2004). 15264904 10.1021/jf040095e

[b7] Lin H. S., Yue B. D. & Ho P. C. Determination of pterostilbene in rat plasma by a simple HPLC-UV method and its application in pre-clinical pharmacokinetic study. Biomed Chromatogr 23, 1308 (2009). 19488981 10.1002/bmc.1254

[b8] Remsberg C. M. . Pharmacometrics of pterostilbene: preclinical pharmacokinetics and metabolism, anticancer, antiinflammatory, antioxidant and analgesic activity. Phytother Res 22, 169 (2008). 17726731 10.1002/ptr.2277

[b9] McCormack D. & McFadden D. Pterostilbene and cancer: current review. J Surg Res 173, e53 (2012). 22099605 10.1016/j.jss.2011.09.054

[b10] Tolba M. F. & Abdel-Rahman S. Z. Pterostilbine, an active component of blueberries, sensitizes colon cancer cells to 5-fluorouracil cytotoxicity. Sci Rep 5, 15239 (2015). 26472352 10.1038/srep15239 PMC4608003

[b11] Suganya N. . Proteomic identification of pterostilbene-mediated anticancer activities in HepG2 cells. Chem Res Toxicol 27, 1243 (2014). 24936659 10.1021/tx5001392

[b12] Wang T. T., Schoene N. W., Kim Y. S., Mizuno C. S. & Rimando A. M. Differential effects of resveratrol and its naturally occurring methylether analogs on cell cycle and apoptosis in human androgen-responsive LNCaP cancer cells. Mol Nutr Food Res 54, 335 (2010). 20077416 10.1002/mnfr.200900143

[b13] Alosi J. A., McDonald D. E., Schneider J. S., Privette A. R. & McFadden D. W. Pterostilbene inhibits breast cancer *in vitro* through mitochondrial depolarization and induction of caspase-dependent apoptosis. J Surg Res 161, 195 (2010). 20031172 10.1016/j.jss.2009.07.027

[b14] Hsiao P. C. . Pterostilbene simultaneously induced G0/G1-phase arrest and MAPK-mediated mitochondrial-derived apoptosis in human acute myeloid leukemia cell lines. PLoS One 9, e105342 (2014). 25144448 10.1371/journal.pone.0105342 PMC4140770

[b15] Roslie H. . 3,5-dibenzyloxy-4′-hydroxystilbene induces early caspase-9 activation during apoptosis in human K562 chronic myelogenous leukemia cells. J Toxicol Sci 37, 13 (2012). 22293408 10.2131/jts.37.13

[b16] Tolomeo M. . Pterostilbene and 3′-hydroxypterostilbene are effective apoptosis-inducing agents in MDR and BCR-ABL-expressing leukemia cells. Int J Biochem Cell Biol 37, 1709 (2005). 15878840 10.1016/j.biocel.2005.03.004

[b17] Khan N., Afaq F. & Mukhtar H. Apoptosis by dietary factors: the suicide solution for delaying cancer growth. Carcinogenesis 28, 233 (2007). 17151090 10.1093/carcin/bgl243

[b18] Kim R., Emi M. & Tanabe K. Role of mitochondria as the gardens of cell death. Cancer Chemother Pharmacol 57, 545 (2006). 16175394 10.1007/s00280-005-0111-7

[b19] Staudt L. M. & Dave S. The biology of human lymphoid malignancies revealed by gene expression profiling. Adv Immunol 87, 163 (2005). 16102574 10.1016/S0065-2776(05)87005-1 PMC1351148

[b20] Lenz G. . Stromal gene signatures in large-B-cell lymphomas. N Engl J Med 359, 2313 (2008). 19038878 10.1056/NEJMoa0802885 PMC9103713

[b21] Hain R., Bieseler B., Kindl H., Schroder G. & Stocker R. Expression of a stilbene synthase gene in Nicotiana tabacum results in synthesis of the phytoalexin resveratrol. Plant Mol Biol 15, 325 (1990). 2103451 10.1007/BF00036918

[b22] Garcia-Zepeda S. P., Garcia-Villa E., Diaz-Chavez J., Hernandez-Pando R. & Gariglio P. Resveratrol induces cell death in cervical cancer cells through apoptosis and autophagy. Eur J Cancer Prev 22, 577 (2013). 23603746 10.1097/CEJ.0b013e328360345f

[b23] Gautam S. C., Xu Y. X., Dumaguin M., Janakiraman N. & Chapman R. A. Resveratrol selectively inhibits leukemia cells: a prospective agent for *ex vivo* bone marrow purging. Bone Marrow Transplant 25, 639 (2000). 10734298 10.1038/sj.bmt.1702189

[b24] Liu P. . Resveratrol induces apoptosis of pancreatic cancers cells by inhibiting miR-21 regulation of BCL-2 expression. Clin Transl Oncol 15, 741 (2013). 23359184 10.1007/s12094-012-0999-4

[b25] Sheth S. . Resveratrol reduces prostate cancer growth and metastasis by inhibiting the Akt/MicroRNA-21 pathway. PLoS One 7, e51655 (2012). 23272133 10.1371/journal.pone.0051655 PMC3521661

[b26] Kapetanovic I. M., Muzzio M., Huang Z., Thompson T. N. & McCormick D. L. Pharmacokinetics, oral bioavailability, and metabolic profile of resveratrol and its dimethylether analog, pterostilbene, in rats. Cancer Chemother Pharmacol 68, 593 (2011). 21116625 10.1007/s00280-010-1525-4 PMC3090701

[b27] Asensi M. . Inhibition of cancer growth by resveratrol is related to its low bioavailability. Free Radic Biol Med 33, 387 (2002). 12126761 10.1016/s0891-5849(02)00911-5

[b28] Nutakul W. . Inhibitory effects of resveratrol and pterostilbene on human colon cancer cells: a side-by-side comparison. J Agric Food Chem 59, 10964 (2011). 21936500 10.1021/jf202846b PMC3201709

[b29] Riche D. M. . Analysis of safety from a human clinical trial with pterostilbene. J Toxicol 2013, 463595 (2013). 23431291 10.1155/2013/463595 PMC3575612

[b30] Chen R. J., Ho C. T. & Wang Y. J. Pterostilbene induces autophagy and apoptosis in sensitive and chemoresistant human bladder cancer cells. Mol Nutr Food Res 54, 1819 (2010). 20603834 10.1002/mnfr.201000067

[b31] Pan M. H. . Pterostilbene inhibited tumor invasion via suppressing multiple signal transduction pathways in human hepatocellular carcinoma cells. Carcinogenesis 30, 1234 (2009). 19447859 10.1093/carcin/bgp121

[b32] Siedlecka-Kroplewska K., Jozwik A., Kaszubowska L., Kowalczyk A. & Boguslawski W. Pterostilbene induces cell cycle arrest and apoptosis in MOLT4 human leukemia cells. Folia Histochem Cytobiol 50, 574 (2012). 23264221 10.5603/20257

[b33] Sherr C. J. Cancer cell cycles. Science 274, 1672 (1996). 8939849 10.1126/science.274.5293.1672

[b34] Lu C. . Serum starvation induces H2AX phosphorylation to regulate apoptosis via p38 MAPK pathway. Febs Lett 582, 2703 (2008). 18619440 10.1016/j.febslet.2008.06.051

[b35] Podhorecka M., Skladanowski A. & Bozko P. H2AX Phosphorylation: Its Role in DNA Damage Response and Cancer Therapy. J Nucleic Acids 2010 (2010). 10.4061/2010/920161 PMC2929501 20811597

[b36] Dai B. . Functional and molecular interactions between ERK and CHK2 in diffuse large B-cell lymphoma. Nat Commun 2, 402 (2011). 21772273 10.1038/ncomms1404 PMC3144586

[b37] Roberti M. . Synthesis and biological evaluation of resveratrol and analogues as apoptosis-inducing agents. J Med Chem 46, 3546 (2003). 12877593 10.1021/jm030785u

[b38] Pan M. H., Chang Y. H., Badmaev V., Nagabhushanam K. & Ho C. T. Pterostilbene induces apoptosis and cell cycle arrest in human gastric carcinoma cells. J Agric Food Chem 55, 7777 (2007). 17696482 10.1021/jf071520h

[b39] Cobb M. H. MAP kinase pathways. Prog Biophys Mol Biol 71, 479 (1999). 10354710 10.1016/s0079-6107(98)00056-x

[b40] Paul S. . Dietary intake of pterostilbene, a constituent of blueberries, inhibits the beta-catenin/p65 downstream signaling pathway and colon carcinogenesis in rats. Carcinogenesis 31, 1272 (2010). 20061362 10.1093/carcin/bgq004 PMC2899944

[b41] Suh N. . Pterostilbene, an active constituent of blueberries, suppresses aberrant crypt foci formation in the azoxymethane-induced colon carcinogenesis model in rats. Clin Cancer Res 13, 350 (2007). 17200374 10.1158/1078-0432.CCR-06-1528

